# Status of Humoral and Cellular Immune Responses within 12 Months following CoronaVac Vaccination against COVID-19

**DOI:** 10.1128/mbio.00181-22

**Published:** 2022-04-27

**Authors:** Wei Zhao, Weixin Chen, Juan Li, Meng Chen, Qin Li, Min Lv, ShanShan Zhou, Shuang Bai, Yali Wang, Lichi Zhang, Peng Zhang, Jian Wang, Qun Zheng, Jiang Wu

**Affiliations:** a Experimental Center for Basic Medical Teaching, School of Basic Medical Sciences, Capital Medical University, Beijing, China; b Beijing Center for Disease Prevention and Control, Beijing Research Center for Preventive Medicine, Beijing, China; c Department of Laboratory, Yanjing Medical College, Capital Medical University, Beijing, China; Dartmouth College

**Keywords:** COVID-19 vaccine, 12 months, neutralizing antibody, T cells, CoronaVac

## Abstract

Understanding immune memory to COVID-19 vaccines is critical for the design and optimal vaccination schedule for curbing the COVID-19 pandemic. Here, we assessed the status of humoral and cellular immune responses at 1, 3, 6, and 12 months after two-dose CoronaVac vaccination. A total of 150 participants were enrolled, and 136 of them completed the study through the 12-month endpoint. Our results show that, at 1 month after vaccination, both binding and neutralizing antibodies could be detected; the seropositive rate of binding antibodies and seroconversion rate of neutralizing antibodies were 99% and 50%, respectively. From 3 to 12 months, the binding and neutralizing antibodies declined over time. At 12 months, the binding and neutralizing antibodies were still detectable and significantly higher than the baseline. Gamma interferon (IFN-γ) and interleukin 2 (IL-2) secretion specifically induced by the receptor-binding domain (RBD) persisted at high levels until 6 months and could be observed at 12 months, while the levels of IL-5 and granzyme B (GzmB) were hardly detected, demonstrating a Th1-biased response. In addition, specific CD4^+^ T central memory (T_CM_), CD4^+^ effector memory (T_EM_), CD8^+^ T_EM_, and CD8^+^ terminal effector (T_E_) cells were all detectable and functional up to 12 months after the second dose, as the cells produced IFN-γ, IL-2, and GzmB in response to stimulation of SARS-CoV-2 RBD. Our work provides evidence that CoronaVac induced not only detectable binding and neutralizing antibody responses, but also functional SARS-CoV-2-specific CD4^+^ and CD8^+^ memory T cells for up to 12 months.

## INTRODUCTION

COVID-19 is a worldwide emergency (1). The urgent need for safe and effective interventions to mitigate the global spread of SARS-CoV-2 has prompted international efforts to develop vaccines. As of 8 October 2021, 24 COVID-19 vaccines have been approved for use (2), and more than 6.44 billion doses have been administered (3). However, compared with other vaccines, the time interval between research and development and application of COVID-19 vaccines is very short, and the underlying immunological mechanisms are not well understood, such as antibody persistence, immune memory, etc. Therefore, it is important that more follow-up studies investigate the kinetics of neutralizing antibody and immune memory of T and B cells, which will not only guide the design of vaccination schedules, but also improve the efficacy of vaccines.

CoronaVac (Sinovac Life Sciences, Beijing, China) is an inactivated vaccine against COVID-19, which is currently approved for emergency use in China (4) and has also been included in the World Health Organization’s (WHO) emergency use listing (5). The data derived from phase 1 to 3 trials have shown that inactivated COVID-19 vaccines are effective, immunogenic, and safe in children and adolescents aged 3 to 17 years (6) and adults aged 18 years and older (4). Here, we reported the status of the persistence of antibodies and cellular responses within 12 months after two-doses of CoronaVac.

## RESULTS

### Study design.

**FIG 1:**
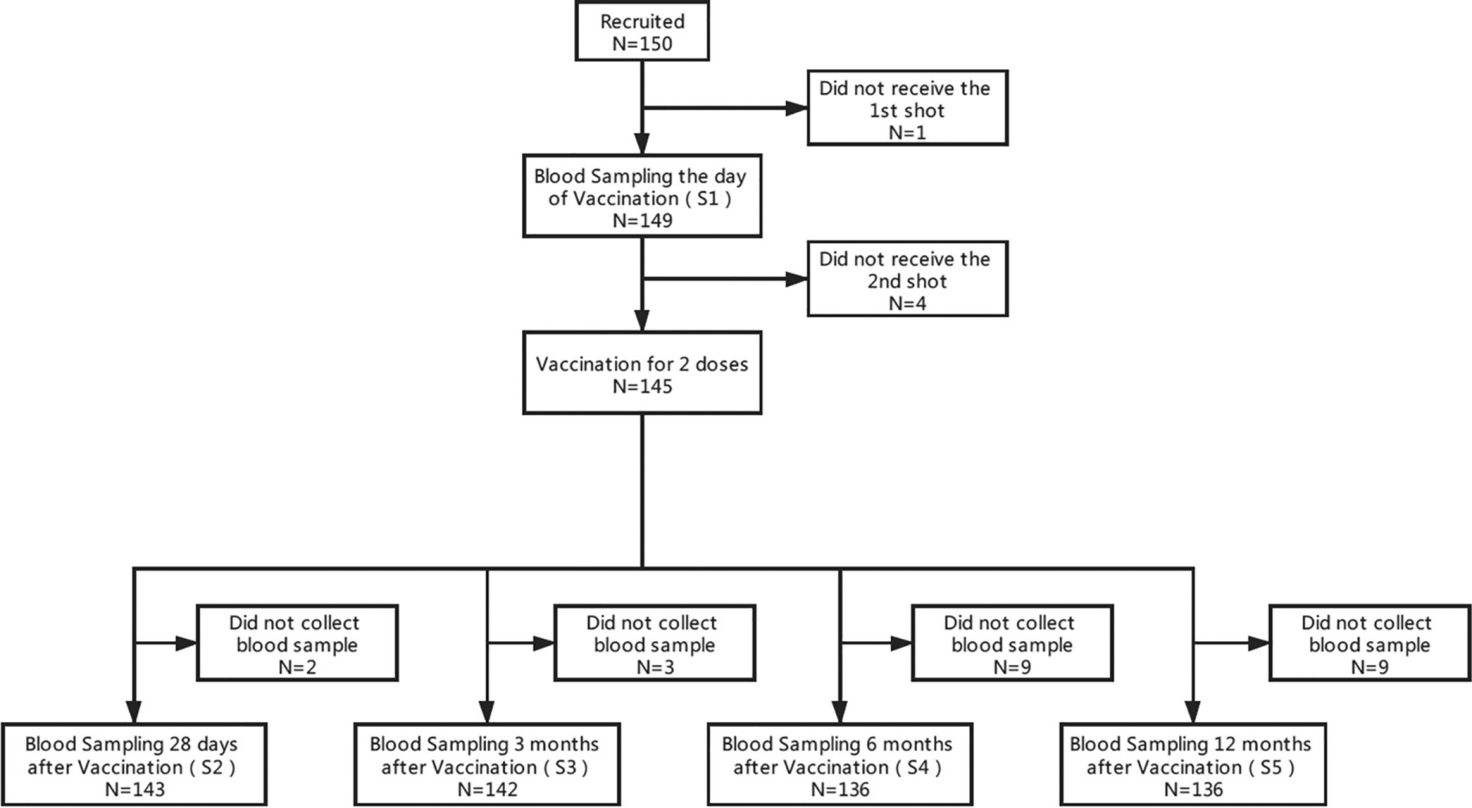
Schedule of sample collection. A total of 150 participants aged 18 to 59 were enrolled in Beijing CDC, China. The participants were administered 3 μg CoronaVac intramuscularly following a 2-shot vaccine schedule, 14 days apart. Following that, the samples, including serum, plasma, and peripheral blood mononuclear cells, were collected on day 0 before vaccination (baseline) and at 1, 3, 6, and 12 months after the second shot. A total of 136 of participants completed the study through the 12-month endpoint.

A total of 150 participants were enrolled this study. Among them, 145 participants received two doses of the investigational product, and 136 participants completed the scheduled visits 12 months after the second shot. The design and schedule of sample collection are shown in Fig. 1.

### Detection of SARS-CoV-2-specific binding antibodies.

**FIG 2:**
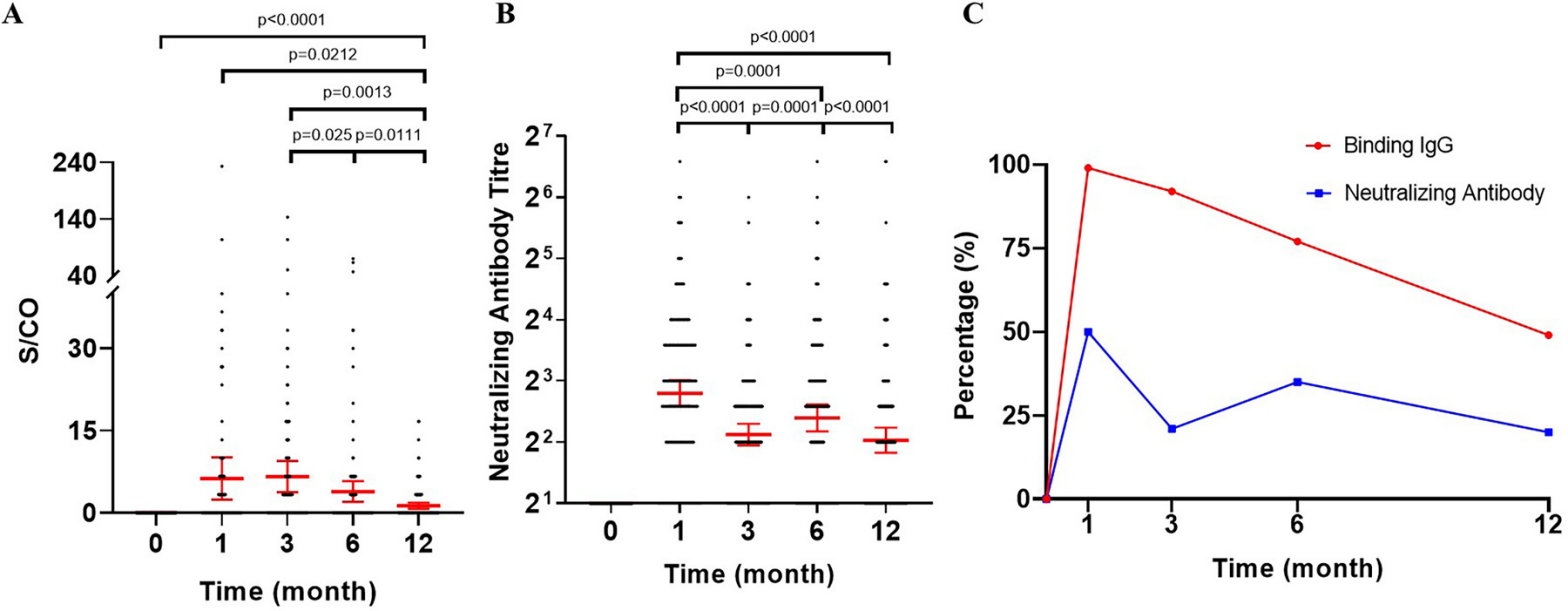
Status of sera IgG and neutralizing antibody response following CoronaVac vaccination. (A and B) Spike RBD-binding IgG (A) and SARS-CoV-2 neutralizing antibody (B) measured by CLIA and microcytopathogenic effect assay. Participants received CoronaVac at day 0 and 14. Each data point represents a serum sample. The error bars of binding antibody are the mean with 95% CI. The error bars of neutralizing antibody are the geometric mean with 95% CI. (C) Seropositive rates of binding IgG and seroconversion rates of neutralizing antibodies were defined as a S/CO value of ≥1.0 and a titer of 8 or higher for neutralizing antibodies to live SARS-CoV-2, respectively. A Wilcoxon matched-pair signed rank test was used for *x*. Two-sided *P* values are shown. CLIA, chemiluminescent immunoassay.

In order to monitor the immunological responses of vaccinees, we collected sequential serum samples (0, 1, 3, 6, and 12 months) from 149 vaccinated health participants. Chemiluminescent immunoassay (CLIA) showed that at baseline, none of the participants had any detectable receptor-binding domain (RBD)-specific IgG antibody (Fig. 2A). At 1 month after the second vaccination, titers of RBD-specific IgG antibodies were strikingly enhanced to a maximum signal-to-cutoff (S/CO) ratio value of 11.26 (95% confidence interval [CI], 9.29 to 13.24), and the seropositive rate was 99% (141 of 143 participants) (Fig. 2A and C). Although the mean concentration of the RBD-specific IgG antibodies at 3 months (S/CO value, 3.87 [95% CI, 2.85 to 4.90]) was only one-third of the peak level observed at the 1 month, the seropositive rate still persisted at a high level (92%, 130 of 142). Thereafter, the antibody titers reached a plateau phase with only a gradual decline from 3 to 12 months (6-month S/CO value, 3.68 [95% CI, 2.43 to 4.94]; 12-month S/CO value, 2.11 [95% CI, 1.50 to 2.72]). The seropositive rates of RBD-specific IgG antibody were 77% (105 of 136) and 49% (67 of 136) at 6 and 12 months after the second vaccination, respectively.

### Neutralizing antibodies against wild-type SARS-CoV-2.

We assessed neutralization activity of sera against live SARS-CoV-2 (virus strain SARS-CoV-2/human/CHN/CN1/2020, GenBank version number MT407649.1) using the microcytopathogenic effect assay, and expressed a 50% inhibitory dilution (ID_50_) (4, 6). As expected, there were no detectable titers of neutralizing antibodies in sera of all study participants at baseline (Fig. 2B). At 1 month after the second vaccination, neutralizing antibody titers increased substantially from baseline to the geometric mean titers (GMT) with a peak level of 7.0 (95% CI, 4.9 to 9.1), while the seroconversion rate was 50% (71 of 143 participants) (Fig. 2B and C). Similar to RBD-specific IgG antibody, at 3 months after the second vaccination, a rapid decline in the GMT of neutralizing antibody (4.4; 95% CI, 2.3 to 6.4) was observed, followed by a plateau phase. Interestingly, the GMT of neutralizing antibody did not decrease continuously at 6 months but increased significantly compared with that at 3 months, reaching 5.3 (95% CI, 3.1 to 7.4). At 12 months, the GMT of the neutralizing antibody decreased to 4.1 (95% CI, 2.0 to 6.2) yet remained significantly higher than the baseline, and there was no significant difference between the GMT of 3 months and 12 months after the second vaccination. The seroconversion rates of neutralizing antibody at 3, 6, and 12 months were 20% (29 of 142), 35% (48 of 136), and 20% (27 of 136), respectively, which were consistent with the changing trend of neutralizing antibody titers.

### Polarization of T-cell responses.

**FIG 3:**
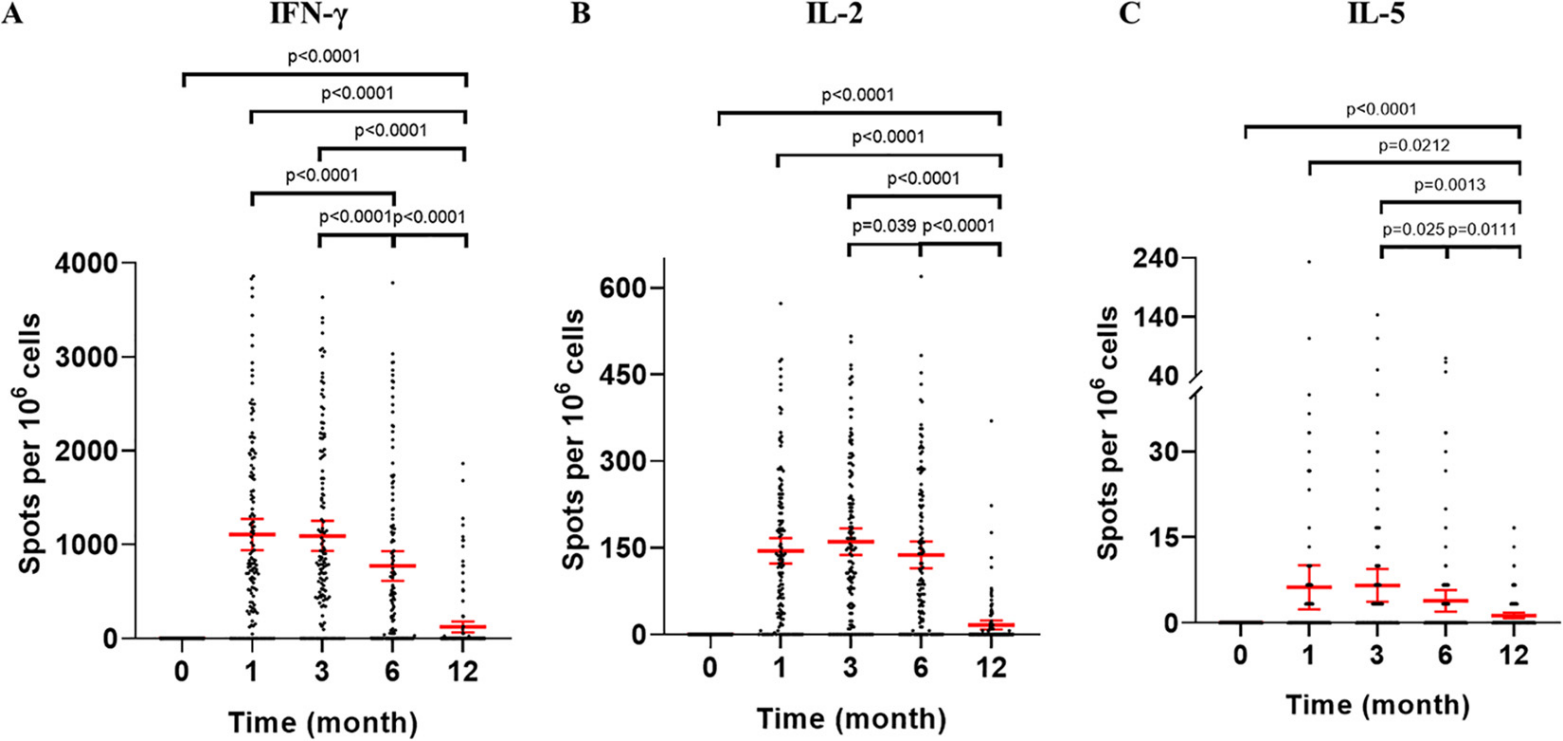
Status of specific T-cell responses following CoronaVac vaccination. (A to C) The numbers of specific T cells with secretion of IFN-γ (A), IL-2 (B), and IL-5 (C) were detected by *ex vivo* ELISpot using isolated PBMC under stimulation with RBD. Each data point represents the mean number of spots from triplicate wells for one participant, after subtraction of the unstimulated control. The error bars are the geometric mean with 95% CI. A Wilcoxon matched-pair signed rank test was used for *x*. Two-sided *P* values are shown. IFN, interferon; IL, interleukin.

SARS-CoV-2 RBD-specific gamma-interferon (IFN-γ), interleukin 2 (IL-2), IL-5, and granzyme B (GrzB) enzyme-linked immunospot (ELISpot) responses were assessed at 1, 3, 6, and 12 months after the second vaccination in peripheral blood mononuclear cells (PBMCs) of all participants (Fig. 3). IFN-γ responses were elicited in participants with a peak frequency (spot-forming cells [SFCs], 1,107.7 [95% CI, 941.1 to 1,274.3]) at 1 month after the second vaccination and stabilized toward 3 months (SFCs, 1,093.1 [95% CI, 931.8 to 1,254.5]) (Fig. 3A). Although some decline in SFCs was seen, relatively high levels of IFN-γ responses persisted to 6 months (SFCs, 772.6 [95% CI, 614.6 to 930.7]). At 12 months, IFN-γ responses declined further but were still detectable (SFCs, 123.3 [95% CI, 64.5 to 182.2]). In addition, IL-2 responses were also noted at each time point after the second vaccination and showed a pattern similar to that of IFN-γ responses; high levels of IL-2 responses persisted until the end of 6 months after the second vaccination (Fig. 3B). Although some participants had detectable IL-5 responses after vaccination (Fig. 3C), IL-5 responses were lower than those of IFN-γ and IL-2 at each time point after vaccination, indicating a Th1-biased cellular immune response. GrzB responses were not detectable at each time point after vaccination.

### Distribution of SARS-CoV-2-specific CD4^+^ and CD8^+^ memory T-cell responses.

**FIG 4:**
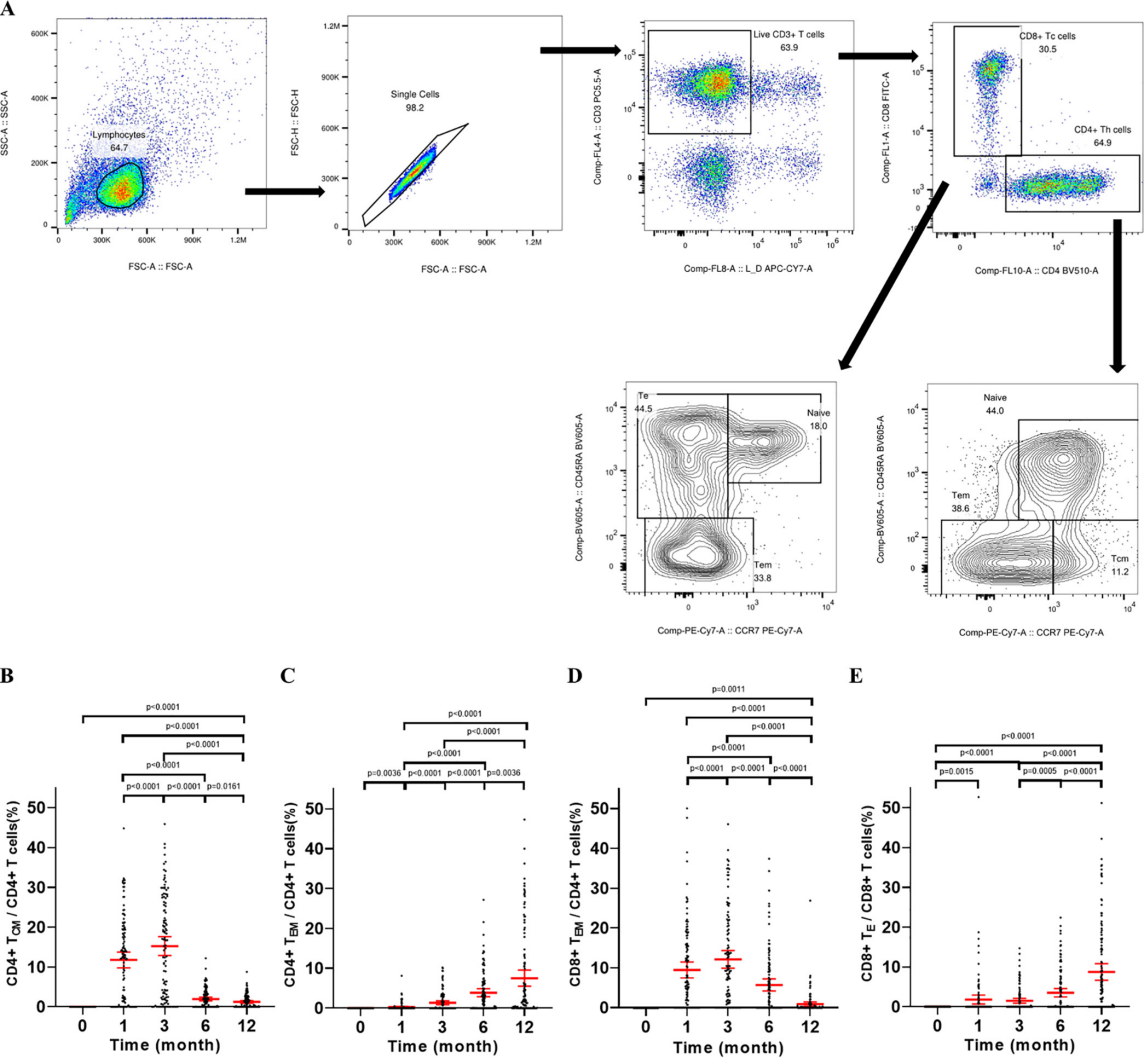
Status of distribution and expression of cytokines by T_CM_ and T_EM_ following CoronaVac vaccination. (A) Example flow cytometry gating strategy. Representative gating of CD3^+^ T cells, CD4^+^ T cells, CD8^+^ T cells, and subsets of CD4^+^ T cells and CD8^+^ T cells. (B and C) Percentage of T_CM_ and T_EM_ of total SARS-CoV-2-specific CD4^+^ T cells. (D and E) Distribution of T_EM_ and T_E_ of total SARS-CoV-2-specific CD8^+^ T cells. The error bars are the geometric mean with 95% CI. A Wilcoxon matched-pair signed rank test was used for *x*. Two-sided *P* values are shown. T_CM_, central memory T cells; T_EM_, effector memory T cells; T_E_, terminal effector T cells.

Memory T-cell subsets and expression of IFN-γ, IL-2, and GrzB were analyzed by using intracellular cytokine staining (ICS) assays to evaluate the SARS-CoV-2 RBD-specific memory T cells in a subset of participants (*n* = 119, in whom sufficient PBMC were available) (Fig. 4). The percentage of RBD-specific CD4^+^ T central memory (T_CM_) cells was significantly higher at 1 month (11.78%) after the second vaccination than that of the baseline, representing 76% (86/113) of participants with detectable RBD-specific CD4^+^ T_CM_ cells (Fig. 4B). Then, the fraction of RBD-specific CD4^+^ T_CM_ cells slightly but significantly increased (15.25%) compared with those of 1 month, declined until 6 months (1.97%), and stabilized toward 12 months (1.24%) after the second vaccination. Conversely, the percentages of subjects with detectable circulating SARS-CoV-2 RBD-specific CD4^+^ T_CM_ cells were 86% (95 of 110), 59% (64 of 108), and 56% (65 of 117) at 3, 6, and 12 months after the second vaccination, respectively. The specific CD8^+^ effector memory (T_EM_) responses were also measured (Fig. 4D). A considerable fraction of RBD-specific CD8^+^ T_EM_ cells was observed at 1 month (9.48%), which peaked at 3 months (12.14%) and thereafter dropped over time (6 months, 5.73%; 12 months, 0.89%). The proportions of subjects with detectable circulating SARS-CoV-2 RBD-specific CD8^+^ effector memory (T_EM_) cells were 69% (78 of 113), 78% (86 of 110), 56% (60 of 108), and 31% (36 of 117) of participants at 1, 3, 6, and 12 months after the last vaccination, respectively. We also observed that the fractions of CD4^+^ effector memory (T_EM_) (Fig. 4C) and CD8^+^ terminal effector (T_E_) (Fig. 4E) cells specific to SARS-CoV-2 RBD increased over time and constituted up to about 7.51% of total peripheral blood CD4^+^ T cells and about 8.74% of total peripheral blood CD8^+^ T cells at 12 months.

### Evaluation of the polyfunctionality of T cells responding to SARS-CoV-2 RBD.

**FIG 5:**
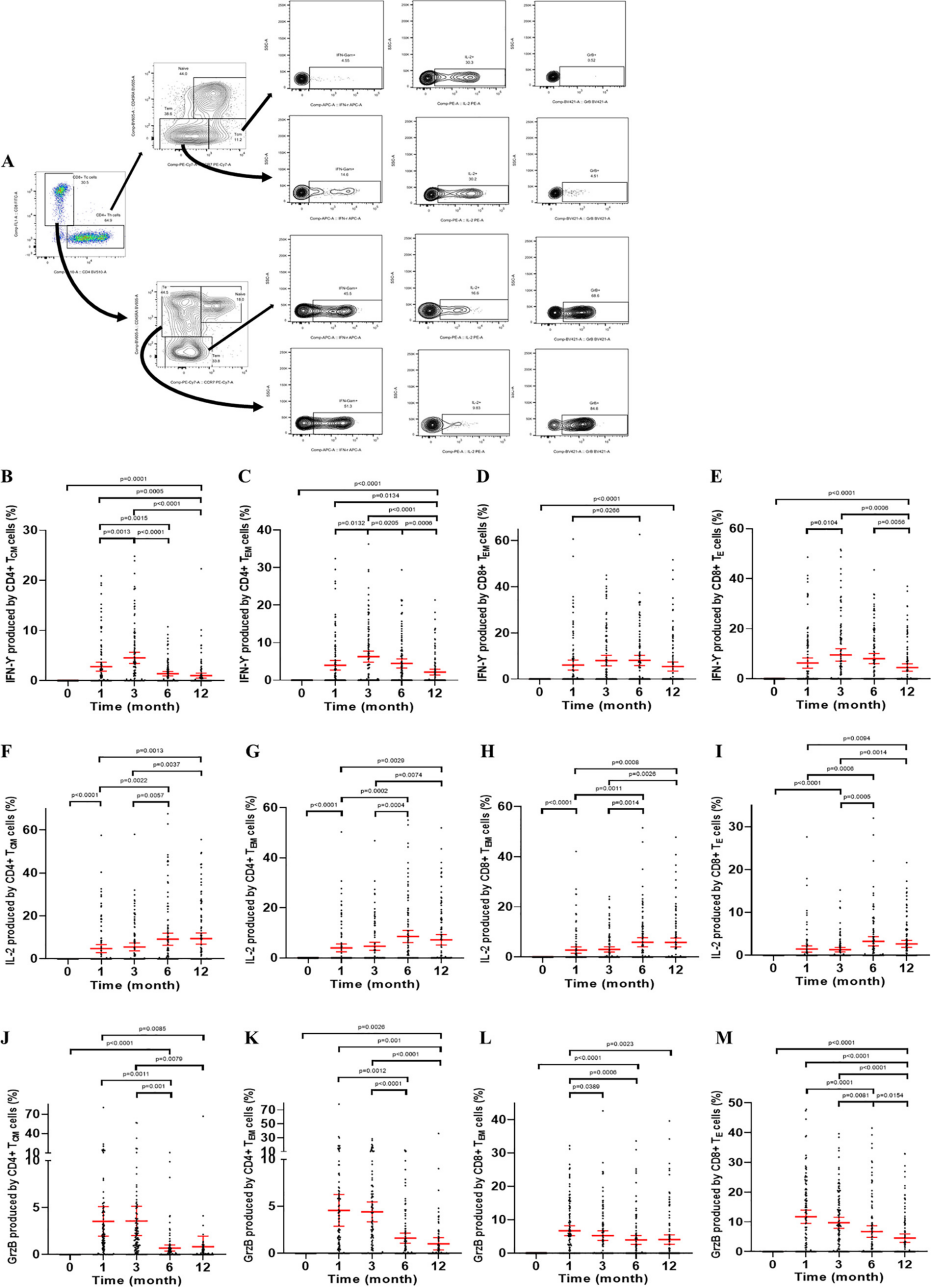
Status of expression of cytokines by CD4^+^ and CD8^+^ memory T cells following CoronaVac vaccination. (A) Example flow cytometry gating strategy. (B to M) Percentages of CD4^+^ T_CM_, CD4^+^ T_EM_, CD8^+^ T_EM_, and CD8^+^ T_E_ cells expressing IFN-γ (B to E), IL-2 (F to I), and GrzB (J to M) that responded specifically to RBD stimulation. The error bars are the geometric mean with 95% CI. A Wilcoxon matched-pair signed rank test was used for *x*. Two-sided *P* values are shown. IFN, interferon; IL, interleukin. T_CM_, central memory T cells; T_EM_, effector memory T cells; T_E_, terminal effector T cells.

Memory T cells rapidly express a wide variety of cytokines upon antigen recognition. To assess the functionality of the SARS-CoV-2-specific memory CD4^+^ and CD8^+^ T-cell responses, we further measured intracellular cytokines expressed by these cells in response to SARS-CoV-2 RBD stimulation. IFN-γ-producing memory CD4^+^ T cells exhibited similar kinetics to IFN-γ-producing memory CD8^+^ T cells, in which IFN-γ production started at 1 month, reached the peak at 3 or 6 months, and thereafter dropped over time (Fig. 5B to E). GzmB is a type of cytotoxic granule produced by NK cells and activated cytotoxic T lymphocytes (CTLs) (7). As expected, the GzmB production by specific memory CD4^+^ T and CD8^+^ T cells increased rapidly at 1 month after the second vaccination, maintained a high percentage to 3 months, and then gradually decreased (Fig. 5J to M). Interestingly, the fraction of CD4^+^ T_CM_, CD4^+^ T_EM_, CD8^+^ T_EM_, and CD8^+^ T_E_ cells producing IL-2 continued to rise from 1 to 6 months after the second dose and maintained a high level throughout the follow-up period (until 12 months) (Fig. 5F to I). As shown in Fig. 5, the SARS-CoV-2-specific CD4^+^ T_CM_, CD4^+^ T_EM_, CD8^+^ T_EM_, and CD8^+^ T_E_ cells were all functional up to 12 months after the second dose, as the cells produced IFN-γ, IL-2, and GzmB in response to SARS-CoV-2-specific RBD. Therefore, CoronaVac is able to elicit not only durable SARS-CoV-2-specific memory CD4^+^T cells, but also SARS-CoV-2-specific memory CD8^+^ T cells.

## DISCUSSION

In the present study, we monitored the 12-month durability of humoral and cellular immune responses in 145 individuals who received two doses of CoronaVac (3 μg/per dose, with an interval of 14 days). Our findings extended previously reported results (4) and showed that the seropositive rate of binding antibodies and seroconversion rate of neutralizing antibodies were 99% and 50% at 1 month, respectively. Although the level of binding and neutralizing antibodies decreased over the sampling period, they were still detectable and significantly higher than the baseline after 12 months. More importantly, the status of robustly expanded SARS-CoV-2 RBD-specific memory CD4^+^ and CD8^+^ T cells in the peripheral circulation were monitored for 12 months post-booster vaccination. Furthermore, ELISpot responses and ICS used to characterize T-cell cytokine responses showed that the profile of cytokine secretion was mainly toward the Th1 (IFN-γ and IL-2) rather than the Th2 (IL-5) pathway, suggesting that CoronaVac predominantly induces Th1-biased cellular immune responses. In addition, it is also worth noting that CoronaVac induced detectable antibody responses as well as cellular immune responses for up to 12 months.

Defining the durability of antibody responses to COVID-19 vaccination is important for understanding COVID-19 disease prevention. However, to our knowledge at the moment, the data on the long-term persistence of antibody responses after COVID-19 vaccination are very limited. It has been shown that the Moderna mRNA-1273 vaccine (100-μg per dose) produces high levels of binding and neutralizing antibodies that declined slightly over time until 90 days after the booster vaccination (8, 9). In addition, a significant trend of waning antibody levels with time has been observed in both AstraZeneca ChAdOx1 and Pfizer BNT162b2, with antibody levels decreasing by about 5-fold for ChAdOx1 and by about 2-fold for BNT162b2, between 21 and 41 days and 70 days or more after the second dose, respectively (10). At 320 days, titers of SARS-CoV-2 spike (S) protein-specific IgG in AstraZeneca ChAdOx1 declined to less than a third of the peak titers, although it remained higher than the baseline after a single dose of 5 × 10^10^ viral particles booster vaccine (11). Numerically, the humoral responses of CoronaVac are not as strong as those of other COVID-19 vaccines; however, it is difficult to directly evaluate the capacity for producing antibodies among different vaccines without a direct comparison due to the heterogeneity of neutralization assays. Even though the same live virus is used for neutralization analysis, the results vary from laboratory to laboratory due to the lack of standardized laboratory methods for SARS-CoV-2 neutralization and experimental procedures, including virus titration, serum dilution, virus-serum neutralization, readout, and reporting methods (12). Additionally, the relatively low humoral responses of CoronaVac in the present study might be associated with the relatively short vaccination schedule used. It has been shown that a more robust antibody response can be generated by the day 0 and 28 vaccination schedule compared to the day 0 and 14 schedule. Therefore, the day 0 and 28 vaccination schedule is the current routine vaccination schedule for CoronaVac (4, 13).

Although recent work has focused on antibody responses, memory CD8^+^ T cells play critical role in defending against viral infection through killing virus-infected cells and expressing relevant cytokines and cytolytic molecules (14). CD8^+^ T-cell responses may also contribute to protection, particularly in the setting of waning or borderline antibody responses (15), or potentially against viral variants that are partially resistant to antibodies (16). Previous studies on severe acute respiratory syndrome (SARS) and Middle East respiratory syndrome (MERS) have shown temporary increases in specific antibodies, and that antibody levels decline quickly in patients after recovery, whereas the specific CD4^+^ and CD8^+^ T-cell responses play an essential role in the control of SARS and MERS (17, 18). Some studies have shown that the reduction in the number of T cells is related to poor clinical outcomes and immune pathogenesis, while adequate T-cell counts and appropriate effector function are associated with patients having mild disease symptoms or successful rehabilitation (19). Grifoni et al. have reported that circulating SARS-CoV-2-specific CD4^+^ and CD8^+^ T cells are 100% and 70%, respectively, in a small group of COVID-19 convalescent patients (*n* = 20) (20). In addition, another study has shown that the percentages of CD4^+^ and CD8^+^ T cells concomitantly increase from day 7 after infection and persist for 7 days as the symptoms disappear (21). In contrast, in the present study we also interrogated the presence of functional CD4^+^ and CD8^+^ memory T cells in participants who received the vaccine. ELISpot results showed that RBD-specific T cells secreting IFN-γ and IL-2 persisted through 12 months after the second vaccination. Meanwhile, SARS-CoV-2 RBD-specific memory CD4^+^ and CD8^+^ T cells still expressed detectable cytokines IFN-γ, IL-2, and GzmB throughout the study duration. Together, these data demonstrate that CoronaVac is able to elicit SARS-CoV-2 RBD-specific memory CD4^+^ and CD8^+^ T cells, while these cells could be maintained and still have the capacity to produce effector cytokines after restimulation 12 months postboost. Moreover, a prominent population of CD4^+^ and CD8^+^ memory T cells were biased toward T_CM_, T_EM_, and T_E_ subsets through 12 months post-booster vaccination. Research has shown that T_EM_ and T_E_ subsets exhibit rapid cytotoxicity to eliminate the infected cells but tend to be more short-lived than T_CM_ (22). Conversely, the T_CM_ subset exhibits superior recall capacity (22). Although the classical immunological theory suggests that the inactivated vaccines are not thought to induce CD8^+^ T-cell responses, our data suggest that the structural integrity of whole SARS-CoV-2 might be the key to elicit antiviral CD8^+^ memory T-cell responses (23). The exact mechanism behind this hypothesis, of course, needs further investigation.

An advantage of inactivated COVID-19 vaccines is that, in addition to S protein, which is the main target of most vaccine efforts, they also contain additional conserved SARS-CoV-2 antigens (24). This means that more epitopes, especially those conserved epitopes in proteins other than S protein, are also engaged in T-cell responses induced by inactivated COVID-19 vaccines compared with mRNA, recombinant protein, or viral vector vaccines involving only RBD or S protein (25, 26). Therefore, in a head-to-head comparison, CoronaVac elicited higher structural protein-specific CD4^+^ and CD8^+^ T-cell responses than Pfizer BNT162b2, due to the presence of additional nucleocapsid (N) and envelope (E) proteins (27). In addition, recent studies have shown that vaccines including AstraZeneca ChAdOx1 (28), Johnson & Johnson Ad26.COV2.S (29), Novavax NVX-CoV2373 (30), and Pfizer BNT162b2 (31) have demonstrated reduced neutralization of SARS-CoV-2 B.1.351 (Beta) variant. SARS-CoV-2 B.1.1.529 (Omicron) variant also seemingly escapes neutralizing antibodies (32, 33). However, specific T-cell responses induced by natural infection or inactivated COVID-19 vaccine have been found to target the epitopes of S, N, and E proteins between ancestral and SARS-CoV-2 variants of concern (VOCs) (20, 26, 34). Since antigenic changes in conserved, internal structural viral proteins that are the primary focus of T-cell responses are rare in VOCs, such inactivated vaccines are less likely to be affected by antibody escape mutations in VOCs (35–37). Thus, inactivated COVID-19 vaccines are expected to be effective against both ancestral and variant SARS-CoV-2.

However, it is notable that there are some limitations. First, in order to compare the immune responses of three commercially approved COVID-19 vaccines (inactivated vaccine, adenovirus-based vaccine, and RBD subunit vaccine) in China, we only tested the T-cell responses to RBD which is contained in all three vaccines. Second, because the participants involved in the study were aged 18 to 59 years, the generalizability to those at risk for SARS-CoV-2 infection and in other regions requires further study. Finally, we did not perform a more in-depth T-cell analysis before and after vaccination due to the limited volumes of blood samples available. These issues are being addressed by the ongoing clinical program.

In conclusion, although the seroconversion rate of neutralizing antibodies was only 50% at 1 month, binding and neutralizing antibodies were still detectable at 12 months after two doses of CoronaVac. It is also worth noting that SARS-CoV-2-specific CD4^+^ and CD8^+^ memory T cells were all functional up to 12 months.

## MATERIALS AND METHODS

### Study design and participants.

The prospective cohort study was performed to evaluate the immunogenicity of an inactivated COVID-19 vaccine (CoronaVac; Sinovac Life Sciences, Beijing, China) in adults aged 18 to 59 years and followed up for 12 months after two vaccinations. Participants who were healthy, nonpregnant adults 18 to 59 years of age were recruited in Beijing, China. All participants provided written informed consent before enrollment. The trial protocol was approved by the Ethics Committee of Beijing CDC (2020-28) and was performed in accordance with the requirements of Good Clinical Practice of China and the International Conference on Harmonisation. The main exclusion criteria included history of SARS-CoV, SARS-CoV-2, or Middle East respiratory syndrome infection, high-risk epidemiology history within 14 days before enrollment (e.g., travel or residence history in communities with case reports, or contact history with someone infected with SARS-CoV-2), axillary temperature of more than 37.0°C, and history of allergy to any vaccine component. A complete list of exclusion criteria is included in the protocol. The participants were administered 3 μg CoronaVac intramuscularly following a 2-shot vaccine schedule, 14 days apart. Following that, the samples, including serum and peripheral blood mononuclear cells, were collected for investigation of exploratory endpoint.

### PBMC and serum collection.

Blood samples were collected from participants on day 0 before vaccination (baseline) and at 1, 3, 6, and 12 months after the second shot for analyzing immunogenicity of vaccination. At time points for immunological analyses, blood samples were taken in both plain and heparinized collection tubes. Samples were processed by the laboratory within 4 h of the blood draw. Plain tubes were processed for the collection of blood serum. Tubes were centrifuged at 1,800 rpm for 5 min, and the serum was harvested for storage at −80°C until required. PBMCs were separated from Ficoll-Paque gradient using 50-mL Leucosep tubes (Greiner Bio-One, Germany) according to the manufacturer’s instructions and frozen at −80°C before being stored in liquid nitrogen. PBMCs were thawed at 37°C and washed twice before use.

### RBD-binding IgG assay.

The commercial chemiluminescence detection kits (2019-nCoV IgG antibody detection kit; Bioscience Diagnostics, Tianjin, China) were employed to measure SARS-CoV-2 RBD-specific IgG following the manufacturer’s instructions as described before (13). The positive cutoff value for RBD-specific IgG antibodies was defined as an S/CO value of ≥1.0.

### SARS-CoV-2 neutralization assay.

The titrates of neutralizing antibodies against live SARS-CoV-2 (virus strain SARS-CoV-2/human/CHN/CN1/2020, GenBank number MT407649.1) were quantified using the micro cytopathogenic effect assay (6). Briefly, serum samples were inactivated at 56°C for 30 min and serially diluted with cell culture medium in 2-fold steps. The diluted serum samples were incubated with equal volumes (50 μL) of the live SARS-CoV-2 virus suspension, with a 50% cell culture infective dose of 100 for 2 h at 37.0°C. Vero cells (1.0 × 10^5^ to 2.0 × 10^5^ cells/mL) were then added to the serum-virus suspensions in microplates in duplicate and incubated at 36.5°C for 5 days. Cytopathic effects were recorded under microscopes, and the neutralizing antibody titer was calculated by 50% infective dose (ID_50_). All procedures related to the virus neutralization test were performed in a level 3 biosafety laboratory. Seroconversion was defined as a change from seronegative at baseline to seropositive or a 4-fold titer increase if the participant was seropositive at baseline. The positive cutoff of the titer for neutralizing antibodies to live SARS-CoV-2 was 1/8 (4, 6, 38).

### ELISpot assay.

ELISpot assays were used to evaluate cellular immune responses through measuring expression of IFN-γ, IL-2, IL-5, and GrzB by PBMC stimulated with RBD according to the manufacturer’s standard protocol (Cellular Technology Limited, Ohio, USA). Plates precoated with specific antibodies were washed with phosphate-buffered saline (PBS) and seeded with unfractionated PBMC at 250,000 cells/well. The wells were plated with unfractionated PBMC at 300,000 cells/well, and the cells were cultured with SARS-CoV-2 RBD at a concentration of 0.2 μg/mL. After incubation at 37°C for 24 h, the cells were removed and the plates processed according to the instructions of the manufacturer. The number of spots was determined automatically with an automatic CTL Immunospot reader (Cellular Technology, Shaker Heights, Ohio). The background was defined as the spots produced in the presence of antigen on day 0 before vaccination. All measurements were subtracted by the background values individually, while the subtracted values were corrected to 0. The results are expressed as the number of SFCs per 1,000,000 cells.

### ICS by flow cytometry.

Flow cytometry (FACSLyric; BD, California, USA) was employed to analyze proportions of the CD4^+^ memory T-cell and CD8^+^ memory T-cell subsets. Furthermore, intracellular production of IFN-γ, IL-2, and GrzB by T cells stimulated with RBD was also analyzed using flow cytometry as previously described (39, 40). Briefly, PBMC was stimulated with SARS-CoV-2 RBD at a final concentration of 0.2 μg/mL for 7 h and then for an additional 4 h with leukocyte activation cocktail (BD GolgiPlug, including 50 ng/mL phorbol myristate acetate [PMA], 1 μM ionomycin, and 1 μg/mL brefeldin A). After stimulation, dead cells were labeled using Live/Dead fixable aqua dye from Invitrogen. Surface markers, including CD3PC5.5, CD4BV510, CD8 FITC, CD45RA BV605, and CCR7 PE-cy7 (eBioscience) were stained. Cells were then washed, fixed with Cytofix/Cytoperm, and stained with GrB BV421, IFN-r APC, and IL-2 PE (eBioscience). All samples were acquired on a FACSLyric (BD Biosciences) flow cytometer and analyzed using FlowJo 10 software. CD4 T cells (CD3^+^CD4^+^), CD8 T cells (CD3^+^CD8^+^), and their subsets were defined as T_CM_, CD45RA^+^CCR7^+^; T_EM_, CD45RA^–^CCR7^–^-; and T_E_, CD45RA^+^CCR7^–^ (41). The background was defined as the T-cell subsets and cytokine responses in the presence of antigen and leukocyte activation cocktail on the day 0 before vaccination. All measurements were background-subtracted individually, while the subtracted values were corrected to 0.

### Statistical analysis.

The sample size for this study was based on practical considerations rather than statistical power calculations. Statistical analyses were conducted with SAS 9.4 and GraphPad Prism 8.0.1. Specific binding antibodies against SARS-CoV-2 RBD were presented as S/CO values with 95% CIs. Neutralizing antibodies were presented as GMTs with 95% CIs. Cellular immune responses were presented as the number of SFCs per 1 million cells or as a proportion of positive responders with 95% CIs. The geometric means and 95% CIs were calculated with log_10_ values of the original data, with subsequent antilog transformation applied. The Wilcoxon matched-pair signed rank test was used to compare the differences between groups. Two-sided *P* values of less than 0.05 were considered significant.
